# A key role for the transporter OAT1 in systemic lipid metabolism

**DOI:** 10.1016/j.jbc.2021.100603

**Published:** 2021-03-27

**Authors:** Jeffry C. Granados, Anisha K. Nigam, Kevin T. Bush, Neema Jamshidi, Sanjay K. Nigam

**Affiliations:** 1 Department of Bioengineering, University of California San Diego, La Jolla, California, USA; 2 Skaggs School of Pharmacy and Pharmaceutical Sciences, University of California San Diego, La Jolla, California, USA; 3 Department of Pediatrics, University of California San Diego, La Jolla, California, USA; 4 Department of Radiological Sciences, University of California Los Angeles, Los Angeles, California, USA; 5 Department of Medicine, University of California San Diego, La Jolla, California, USA

**Keywords:** kidney, drug transport, lipid transport, membrane transport, metabolomics, organic anion transporter, proximal tubule, systems biology, DAG, diacylglycerol, DMI, drug–metabolite interaction, LCFA, long-chain fatty acid, MRP, multidrug resistance associated protein, OAT, organic anion transporter, PUFA, polyunsaturated fatty acid, SLC, solute carrier, SLC22, solute carrier 22

## Abstract

Organic anion transporter 1 (OAT1/SLC22A6) is a drug transporter with numerous xenobiotic and endogenous substrates. The Remote Sensing and Signaling Theory suggests that drug transporters with compatible ligand preferences can play a role in “organ crosstalk,” mediating overall organismal communication. Other drug transporters are well known to transport lipids, but surprisingly little is known about the role of OAT1 in lipid metabolism. To explore this subject, we constructed a genome-scale metabolic model using omics data from the *Oat1* knockout mouse. The model implicated OAT1 in the regulation of many classes of lipids, including fatty acids, bile acids, and prostaglandins. Accordingly, serum metabolomics of *Oat1* knockout mice revealed increased polyunsaturated fatty acids, diacylglycerols, and long-chain fatty acids and decreased ceramides and bile acids when compared with wildtype controls. Some aged knockout mice also displayed increased lipid droplets in the liver when compared with wildtype mice. Chemoinformatics and machine learning analyses of these altered lipids defined molecular properties that form the structural basis for lipid-transporter interactions, including the number of rings, positive charge/volume, and complexity of the lipids. Finally, we obtained targeted serum metabolomics data after short-term treatment of rodents with the OAT-inhibiting drug probenecid to identify potential drug–metabolite interactions. The treatment resulted in alterations in eicosanoids and fatty acids, further supporting our metabolic reconstruction predictions. Consistent with the Remote Sensing and Signaling Theory, the data support a role of OAT1 in systemic lipid metabolism.

Studies relating to the systems biology of solute carrier (SLC) and ABC transporter families have suggested that members of the solute carrier 22 (SLC22) family, including organic anion transporters (OATs), organic cation transporters, and organic carnitine/zwitterion transporters, play a central role in endogenous metabolism (1). SLC22 transporters are expressed across nearly all epithelia (*e.g.*, kidney, liver, retina, placenta, olfactory epithelium) and some other tissue types (*e.g.*, blood–brain barrier).

In a network analysis involving over 50 SLC families, SLC22 appears to be one of the main hubs of all SLC families, which together consist of over 400 transporters (2). SLC22 also appears to form a central cluster in a gut–liver–kidney centered cross-tissue coexpression network aimed at clarifying endogenous interactions of SLC, ABC, and drug metabolizing enzymes families (3). SLC22 transporters are highly evolutionarily conserved across several species (4), which suggests a central physiological role. Of note, knockdowns of many SLC22 transporters in fly lead to phenotypes affecting development, cell function, or response to oxidative stress (5).

The systems biology and evolutionary analyses lead to the following question: What are the key functions of this large and highly conserved transporter family in endogenous physiology? Historically, this question has received little attention, in part because several multi-specific SLC22 transporters are among the best known “drug” and toxin transporters; they are responsible for the distribution and elimination of hundreds of clinically relevant xenobiotics.

Here, we focus on organic anion transporter 1 (OAT1/SLC22A6), originally identified as novel kidney transporter (6). It is expressed on the basolateral side of the renal proximal tubule and displays high affinity for antivirals, nonsteroidal anti-inflammatory drugs, diuretics, statins, antihypertensives, and other organic anion drugs (7). Along with OAT3 (SLC22A8), these two transporters account for the bulk of the multispecific organic anion uptake by the kidney proximal tubule, the key part of the nephron involved in drug and toxin handling (8). Therefore, their contribution(s) to drug pharmacokinetics and regulation of systemic metabolism is potentially immense.

In addition to drug transport, a combination of *in vitro*, *ex vivo*, and *in vivo* studies has made it clear that OAT1 and other SLC22 transporters are also involved in regulating endogenous metabolism through modulating the serum levels of key metabolites and signaling molecules (1, 9, 10). OAT1 has been shown to be a contributor to the transport of drugs, toxins, metabolites, signaling molecules, and gut microbe products from the blood into the proximal tubule cells; this is generally believed to lead to excretion into the urine *via* apical membrane drug transporters (7, 11, 12, 13). There is a growing appreciation of how—even in patients treated with a course of antibiotics, or chronically with a small number of drugs for, say, mild hypertension and hyperlipidemia—drugs, metabolites, toxins, and signaling molecules might compete for transport by the OATs and other multispecific SLC and ABC transporters (14). Nevertheless, there is still relatively little understanding of how drug transporters affect *in vivo* metabolism.

It has been posited that a major role of drug transporters is to help maintain local and systemic homeostasis through the regulation of small molecule levels (15, 16). The Remote Sensing and Signaling Theory focuses on the role of drug transporters and drug metabolizing enzymes in mediating intra-organ, inter-organ, and inter-organismal communication partly *via* the transport and metabolism of key metabolites and signaling molecules (3, 9, 15, 16, 17, 18). In this framework, the regulated expression of several transporters with compatible ligand preferences across multiple organs can lead to “organ cross talk” through shared substrates. For example, OAT1 shares metabolites and signaling molecule substrates with multidrug resistance associated proteins (MRPs) and organic anion transporting polypeptides in the liver and elsewhere (19, 20). Within the kidney, proteomic studies have also shown that the abundance of OAT1 is highly correlated with the efflux transporters MRP2 and MRP4 across several species (21). Furthermore, OAT1 is expressed in the placenta and the choroid plexus, albeit at lower levels than in the kidney, implying a role in the function of these organs. Many other clinically relevant SLC and ABC multispecific transporters are also expressed in multiple tissues.

In contrast to ABC transporters, which have been known for some time to play key roles in lipid transport (22) (as well as some metabolic diseases involving lipids), the role of SLC transporters, particularly multispecific ones such as OAT1, in lipid regulation is not well understood. In studies generally focused on other aspects of OAT function, *in vitro* assays have shown that OAT1-overexpressing cells transport certain fatty acids (23, 24). Nevertheless, there has been surprisingly little specific attention given to the role of OAT1 in lipid regulation.

Although metabolic reconstructions centered on the OATs have been performed in the past (24, 25, 26), genome-scale metabolic reconstruction methods using omics data have advanced considerably in recent years and now include several hundred more lipid metabolites. To further analyze the role of OAT1 in the regulation of lipids, we generated a novel metabolic reconstruction based on transcriptomic and metabolomic data from the *Oat1* KO mice. The updated model placed an even greater emphasis on the lipids affected by OAT1 than previous reconstructions. Experimentally, targeted metabolomics analyzing hundreds of endogenous metabolites in the serum of *Oat1* KO mice showed that the absence of this transporter had a striking impact on many different aspects of lipid metabolism. Many of the altered metabolites are well-established signaling molecules known to regulate G-protein coupled receptors and nuclear receptors in remote organs (27, 28). Because of the large number of lipid metabolites with varying chemical structures analyzed, we were able to combine a chemoinformatics approach with machine learning methods to define distinct molecular properties of lipids regulated by OAT1. This work not only highlights the potential of OAT1 in the modulation of systemic lipid metabolism and signaling, but, as we show, it also provides insight into certain drug-induced dyslipidemias such as those seen with chronic treatment with OAT1-transported diuretics or antiviral cocktails (29).

## Results

### Metabolic reconstructions based on omics data from the Oat1 KO mice predict alterations in lipid metabolism

In recent years, genome-scale metabolic reconstructions have greatly advanced (*e.g.*, Recon3D) and found increasing use in the reconstruction of metabolism when a gene is deleted (30, 31). They are based in transcriptomics data and may be constrained by metabolomics data, when available, to add *in vivo* relevance. Older metabolic reconstructions of *Oat1* knockout animals had linked its absence to several unique metabolic pathways (24, 25), but these models used early methods and databases that were limited by the number of reactions and metabolites present in the genome-scale metabolic model. Often described as the most comprehensive metabolic network model to date, Recon3D has improved upon the previous genome-scale metabolic models and accounts for considerably more biochemical reactions and metabolites from the BiGG database than earlier versions of Recon (32). Particularly relevant to the work presented here is the fact that Recon3D includes over 600 new lipid metabolites (31). Thus, we used Recon3D and focused on lipids, which had only received cursory attention in previous *Oat1* KO metabolic reconstructions.

**Figure 1:**
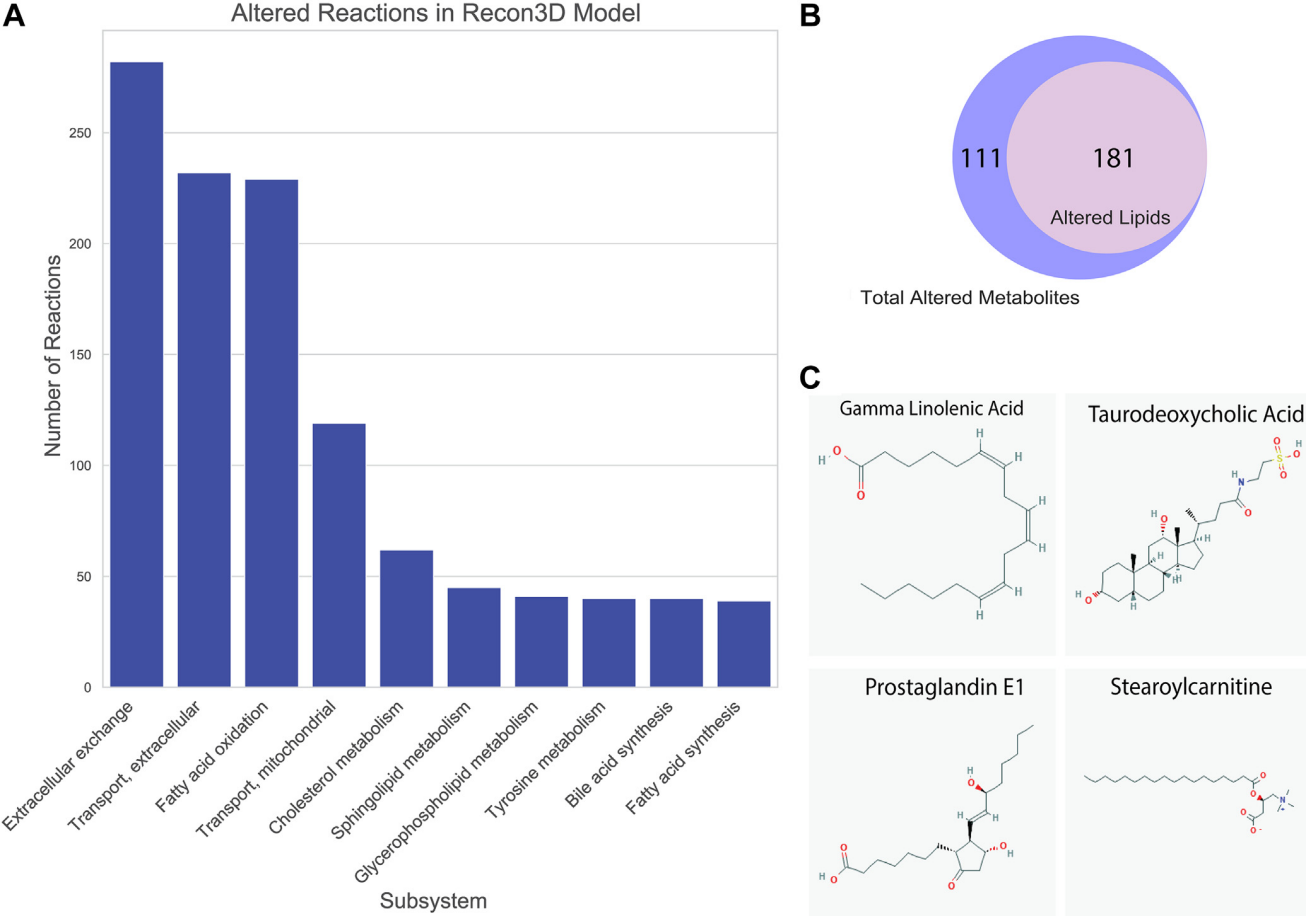
**The altered reactions predicted by the metabolic reconstructions cover a wide range of biological processes.***A*, each altered reaction belongs to a subsystem. The most common subsystem is extracellular exchange, but among the 86 unique subsystems, many lipid-related subsystems are among the most frequent. *B*, of the 312 metabolites predicted to be altered by the reactions, 181 of those are classified as lipids. *C*, polyunsaturated fatty acids, bile acids, prostaglandins, and carnitine derivatives are among the predicted altered lipids, indicating that among the lipids are structurally varied metabolites with different physiological functions.

Wildtype and *Oat1* KO-based metabolic models were constructed using existing transcriptomic and metabolomic data from the *Oat1* KO mice. In these Recon3D models, 1945 reactions belonging to different subsystems were significantly altered (Fig. 1*A*, Table S1). These reactions affected 312 metabolites (Table S2). It is noteworthy that, of those metabolites, 181 (58%) (Table S3) were annotated as lipids by Recon3D subsystems. These included bile acids, fatty acids, eicosanoids, and several other classes of lipid metabolites (Fig. 1*B*).

These alterations in lipid metabolism were much greater than expected, and multiple classes of lipids were predicted to be altered by the new metabolic reconstruction because of OAT1 loss/inhibition (Fig. 1*C*). Thus, this systems biology integration of existing omics data from the *Oat1* KO mouse clearly suggested that regulation/modulation of lipid metabolism, heretofore largely neglected, is a major function of OAT1.

### Lipids are altered in the serum of Oat1 KO mice

Although early untargeted metabolomics data were used to partly constrain the Recon3D metabolic reconstruction, those data were limited to less than two dozen metabolites (13). In the decade since, metabolomics methods and databases have substantially improved, and the number of unique lipids that can be detected and clearly identified is much greater. Because the metabolic reconstructions strongly supported the importance of OAT1 in regulating/modulating lipid metabolites and biochemical reactions involving lipids, targeted metabolomics data from the serum of *Oat1* KO mice and their wildtype controls were used to analyze over 700 endogenous metabolites.

**Figure 2:**
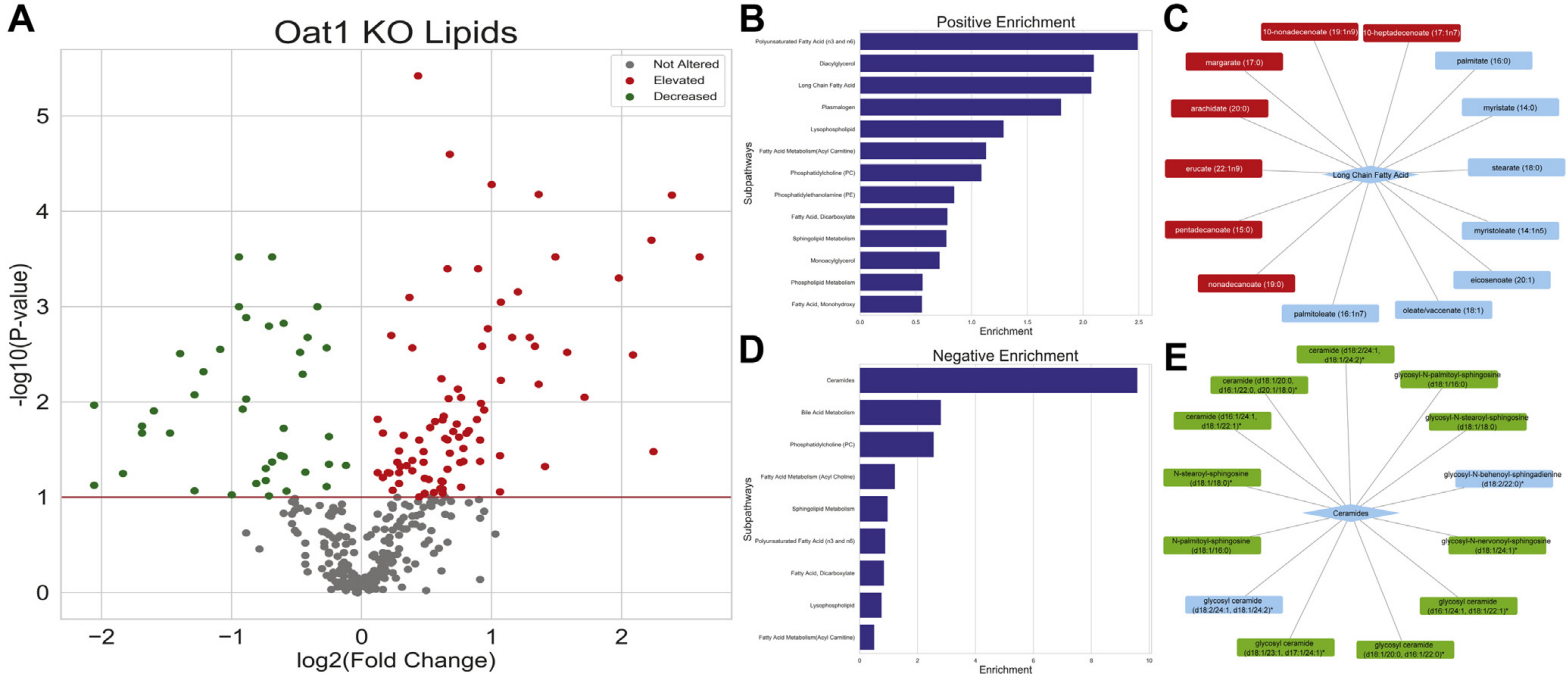
**The lipid profile of the *Oat1* KO differs from the wildtype controls.***A*, among the 342 lipids measured, 126 are significantly altered. There were 86 elevated and 40 decreased representing multiple subpathways. *B*, multiple pathways are enriched for significantly altered metabolites with positive fold changes. *C*, each subpathway contains unique metabolites. The long-chain fatty acid subpathway contains 14 metabolites, and seven are significantly elevated. Metabolites in *red* are significantly elevated, and those in *blue* are not significantly altered. *D*, the loss of OAT1 also results in some pathways being enriched for decreased metabolites. *E*, the ceramide subpathway has 11 of 13 metabolites (*green*) significantly decreased.

Examination of the altered serum metabolites alone revealed at least some involvement of OAT1 in each of eight “Superpathways” associated with the following metabolic functions: Amino Acid, Carbohydrates, Energy, Lipid, Xenobiotics, Peptides, Nucleotide, and Cofactors and Vitamins. Of the 731 metabolites detected on the platform, 342 were classified as lipids (Table S4). It is remarkable that in the *Oat1* knockout, 126 of these metabolites were either significantly altered (*p* ≤ 0.05) or trending toward significantly altered (0.05 < *p* ≤ 0.10). Of these, 86 were elevated and 40 were decreased (Fig. 2*A*). The altered metabolites also covered a wide range of subpathways.

Thus, consistent with the Recon3D omics-based metabolic reconstruction described above, lipid metabolism was by far the most affected superpathway, at least based on the metabolites represented on the platform. We next delved more deeply into the different lipid classes. Then, taking advantage of the large number of altered lipids and their structural diversity, we used chemoinformatics analyses and machine learning methods to identify the molecular and physiochemical properties of the lipids that appear to target a lipid or lipid class for regulation or modulation by OAT1.

### Polyunsaturated fatty acids, long-chain fatty acids, and diacylglycerols are systemically elevated

Enrichment analysis showed that different subpathways were impacted depending on the fold change of the metabolites (Fig. 2, *B* and *D*). Among the 86 elevated metabolites, 32 belonged to either the polyunsaturated fatty acids (PUFAs), long-chain fatty acids (LCFAs), or diacylglycerols (DAGs) subpathway. Eleven (58%) of the 19 metabolites comprising the PUFA subpathway, 7 (50%) of the 14 LCFAs (Fig. 2*C*), and 14 (48%) of the 29 measured DAGs were significantly elevated in the *Oat1* KO. These were the main altered pathways, but several other metabolites, including those in the sphingolipid metabolism and fatty acid metabolism subpathways, also showed significant increases in the serum (Table S4). Elevated metabolites seem likely to be substrates of OAT1, as they are unable to enter OAT1-expressing cells, and thus accumulate in the serum.

### Bile acids and ceramides are systemically decreased

Considering the similarities in pharmaceutical substrates of OAT1 and OAT3, it is quite striking that the levels of bile acids change in opposite directions (33). In the *Oat1* KO mice, bile acids were among the metabolites with the greatest fold changes (Table S4). Taurocholate, tauro-beta-muricholate, tauroursodeoxycholate, and taurodeoxycholate all displayed significant decreases in the serum of the *Oat1* KO with 0.24-, 0.36-, 0.31-, and 0.33-fold changes compared with wildtype mice, respectively.

Of 13 ceramides, 11 were significantly or trending toward significantly decreased in the serum of the *Oat1* KO (Fig. 2*E*). At present, there is little evidence to suggest that these lipids are directly transported by the OATs, and structurally they seem quite different from known OAT substrates (34). Nonetheless, the results suggest that these pathways may somehow be affected by organic anion transporters.

### Chemoinformatics and machine learning methods identified molecular properties separating elevated and decreased lipids

The metabolites associated with a particular superpathway are determined by their functionality in canonical biochemical pathways rather than chemical structures of the lipid metabolites themselves. However, the interaction of individual metabolites themselves with OAT1, a multi-specific transporter, depends on the molecular and physicochemical properties that arise from the structures of the individual lipid metabolites. It has been previously shown that, although elevation of a metabolite in the *Oat1* knockout mouse is not necessarily an indication of direct interaction with the transporter, when a large number of such *in vivo* metabolites are considered, chemoinformatics analyses of altered metabolites together with machine learning classification corresponds well with *in vitro* data and can even make reasonable predictions of OAT1 drug binding and/or transport (34).

Although the variety of structures (*e.g.*, long-chain fatty acids, ceramides, bile acids, carnitine derivatives) in the >100 lipid metabolites might seem difficult to analyze structurally, a chemoinformatics approach, focused on capturing a variety of molecular and physiochemical properties (features) of the lipid metabolites elevated or decreased in the *Oat1* KO, would help to better define the chemical basis of *in vivo* interaction of specific lipid molecules with the transporter. This might prove useful for understanding dyslipidemias that could result from drug–metabolite interactions (DMIs) at the level of the OAT1 transporter.

**Figure 3:**
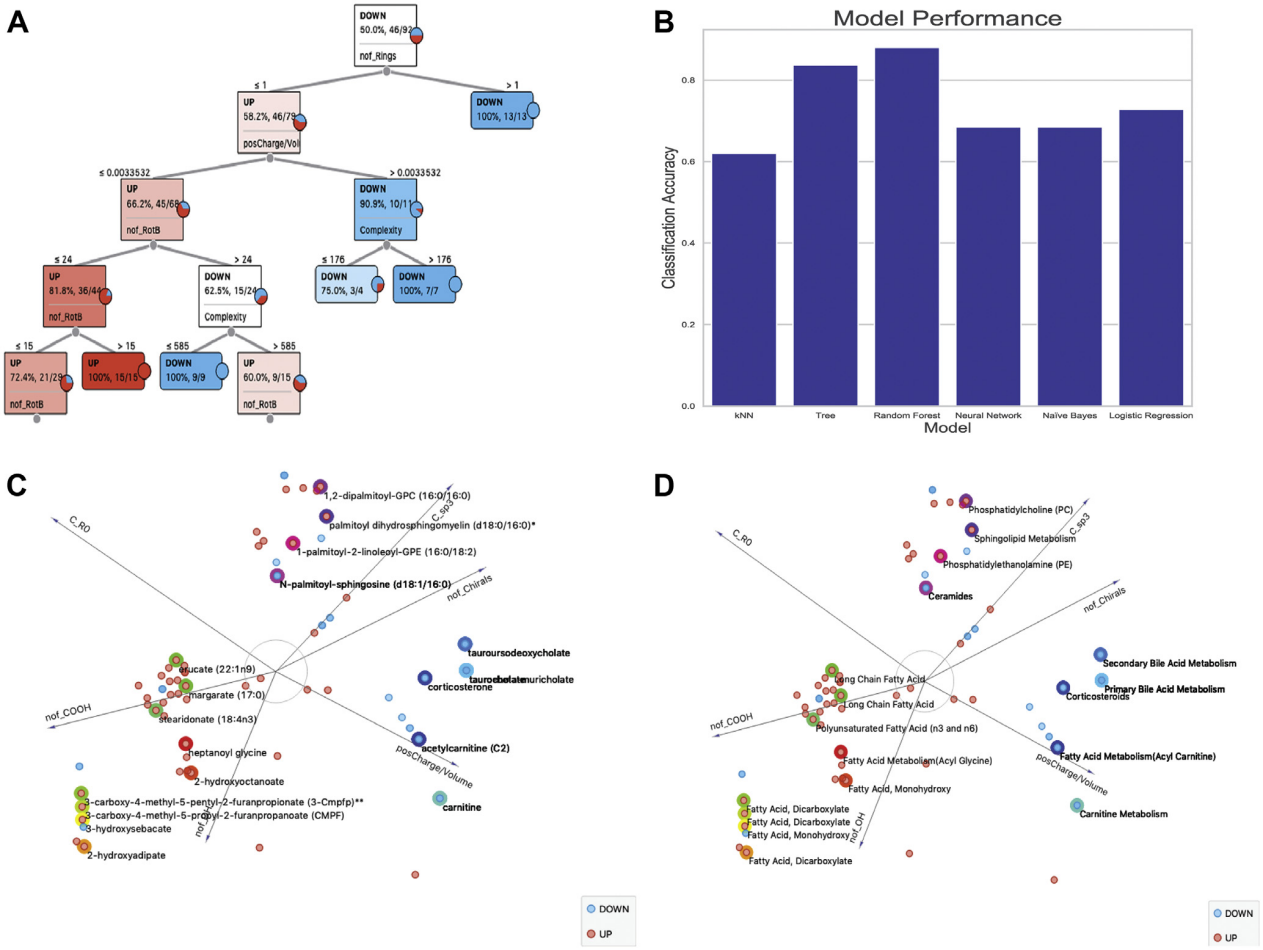
**Chemoinformatics and machine learning analyses help in separating elevated and decreased lipids by physiochemical features.***A*, the decision tree shows four molecular features (number of rings, positive charge over volume, number of rotatable bonds, complexity) that are able to separate elevated and decreased lipids. *B*, different machine learning models achieve classification accuracies up to 85%, with the random forest model having the highest score. *C*, the FreeViz visualization, which is independent of the machine learning analyses, reveals six molecular features (nof_Chirals, C_R0, C_sp3, posCharge/Volume, nof_COOH, nof_OH) that separate the elevated and decreased lipids in the *Oat1* KO. Each metabolite is represented by a point with its name. *D*, each metabolite in the FreeViz plot is represented by its subpathway instead of its name.

We therefore calculated over 30 molecular descriptors for the elevated and decreased lipids in the *Oat1* KO. These properties were then used to determine sets of features that are characteristic for the elevated compared with the decreased group. Visualization of the data as distributions suggested some important differences between the elevated and decreased *Oat1* KO lipids, such as the number of rings. The 32 features were eventually narrowed down to six features. Multiple sets of six features gave reasonable classification accuracy scores with a variety of machine learning methods in the range of 65% to 85% of *Oat1* KO elevated *versus* decreased lipids with acceptable levels of misclassification in a confusion matrix (Fig. S1). A typical decision tree provides insight into how various combinations of these molecular properties can be used to classify *Oat1* KO elevated *versus* decreased lipids with high accuracy (Fig. 3*A*).The actual results depended upon the model (*e.g.*, decision tree, Random Forest, k-nearest neighbors, logistic regression) and the sampling method used to balance the dataset (Fig. 3*B*).The feature set analyzed most often was the following: number of rings (nof_Rings), positive charge/volume, complexity, number of carboxyl groups (nof_COOH), number of hydroxyls (nof_OH), and number of rotatable bonds (nof_RotB). Another set that seemed to give roughly similar classification accuracies and confusion matrices was number of chirals (nof_Chirals), number of carbons with sp3 hybridization (C_sp3), positive charge/volume, nof_COOH, nof_OH, and number of carbons outside of a ring structure (C_R0) (Fig. 3*C* and *D*). For the most part, the misclassified lipids did not belong to a particular subpathway, supporting the notion that the classification was not biased toward accurately classifying a certain type of lipid.

Of note, the following molecular and physiochemical properties were most associated with the *Oat1* KO elevated lipids: C_R0, nof_RotB, nof_COOH, nof_OH. These are consistent, for example, with the abundance of long-chain fatty acids in the elevated group. The decreased group was associated with the nof_Rings and positive charge/volume, which is consistent with the presence of bile acids and ceramides in this group.

### Oat1 KO livers from aged mice show a trend toward accumulation of lipid droplets

Owing to the number of lipids elevated in the serum, we sought to determine if these metabolites were accumulating in the liver, as has sometimes been seen in rodent models with altered serum lipids (35). No histological differences between wildtype and knockout mice were apparent by H&E staining. In younger mice, there was no difference in Oil Red O staining between wildtype and knockout. However, some very aged knockout mouse livers showed increased lipid accumulation and larger droplet size when compared with the wildtype. Only a limited number of very aged *Oat1* knockout mice (∼24-month-old) were available. Three of five of the 24-month-old *Oat1* KO mice livers had visible lipid staining and had larger droplets than the 24-month-old wildtype mice livers (Fig. S2). Nevertheless, it is important to note that not all mice had lipid droplets and the differences did not reach statistical significance. We emphasize that a considerably larger study in aged *Oat1* knockout mice would be needed to statistically support the apparent trend.

### Serum eicosanoid and fatty acid levels are impacted by an OAT-inhibiting drug

Although many substrates that are potentially transported or affected by OAT1 were determined through knockout metabolomics, it is possible that germ-line knockout animals have developed compensatory mechanisms to maintain their health. This may be why, despite their high lipid levels, the *Oat1* KO mice have not (except for what is noted in the previous section) been found to have any tissue alteration, aberrant overall physiology, or decreased life expectancy (10). To better model clinically relevant short-term DMIs, we exposed animals to the high-affinity OAT1-binding, uricosuric drug probenecid and studied its impact on serum eicosanoids, which were largely ignored in the knockout mice metabolomics studies, and free fatty acids. A number of transporters of the prototypical organic anion probe para-aminohippurate have been reported to interact with probenecid *in vitro*; of these, OAT1 appears to be the main target *in vivo* (10).

Most eicosanoids stem from the breakdown of PUFAs, many of which were elevated in the knockout mouse model. In addition, OAT1 has been shown to transport prostaglandin E2, a vasodilator and noradrenaline inhibitor that is a downstream product of arachidonic acid metabolism (36). Thus, there is potential for OAT1 to interact with other eicosanoids and prostaglandins.

**Figure 4:**
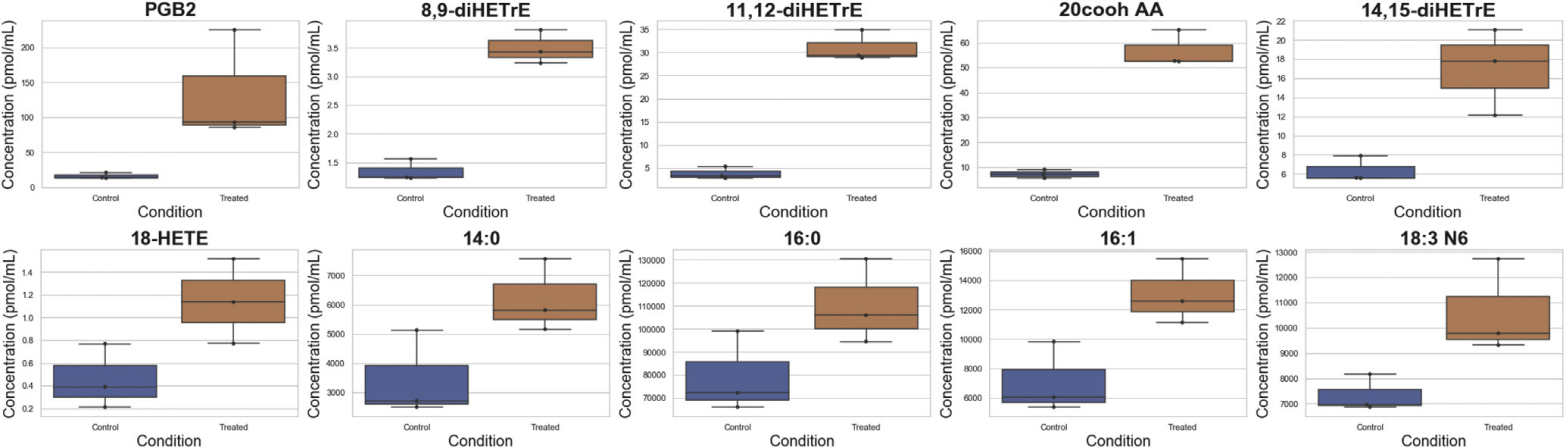
**Ten eicosanoids, prostaglandins, and fatty acids were elevated following probenecid treatment in rats.** Following short-term treatment of wildtype rats with probenecid, an OAT inhibitor, these ten metabolites have increased concentrations in the serum. 8,9-diHETrE, 8,9-dihydroxyeicosatrienoic acid; 11,12-diHETrE, 11,12-dihydroxyeicosatrienoic acid; 20cooh AA, 20-carboxy-arachidonic acid; 14,15-diHETrE, 14,15-dihydroxyeicosatrienoic acid; 18-HETE, 18-hydroxyeicosatetraenoic acids; 14:0, myristic acid; 16:0, palmitic acid; 16:1, palmitoleic acid; 18:3 N6, gamma-linolenic acid; PGB2, prostaglandin B2.

A total of 77 eicosanoids of known identity were detected (Table S5). Six of these metabolites, 11,12-diHETrE (11,12-dihydroxy-5Z,8Z,14Z-eicosatrienoic acid), 20cooh AA, 14,15-diHETrE (14,15-dihydroxy-5Z,8Z,11Z-eicosatrienoic acid), 8,9-diHETrE (8,9-dihydroxy-5Z,11Z,14Z-eicosatrienoic acid), Prostaglandin B2, and 18-HETE, were significantly increased or trending toward significantly increased (*p* ≤ 0.10) after probenecid treatment (Fig. 4). All of these are derived from arachidonic acid, suggesting that OAT1 may regulate the retention and clearance of arachidonic acid breakdown products, a notion consistent with older renal physiology studies performed prior to the discovery of OAT1 (37). PGB2 (15S-hydroxy-9-oxo-5Z,8(12),13E-prostatrienoic acid) was the only prostaglandin to be elevated in the probenecid-treated animals, with an 8.51-fold increase when compared with the control. Although our primary focus is on elevated compounds because of the clear mechanism, it is worth noting that 44 (57%) of the measured eicosanoids were found to be significantly decreased. That these eicosanoids were altered owing to inhibition by probenecid raises the possibility that an important, yet underappreciated, class of DMI (potentially affecting major signaling pathways) is mediated by OAT-transported drugs and includes many eicosanoids and at least one prostaglandin.

Furthermore, we measured 32 fatty acids (Table S6) in the serum samples, and nine were significantly altered or trending toward significance in the probenecid-treated samples. Of those nine fatty acids, four fatty acids, 16:1 (palmitoleic acid), 18:3 N6 (gamma-linolenic acid), 14:0 (myristic acid), and 16:0 (palmitic acid), show significantly elevated levels (Fig. 4). Palmitoleic acid has previously been shown to interact with OAT1 *in vitro*, and gamma-linolenic acid was elevated in the serum of the knockout mice (24).

### Various lipids can inhibit OAT1 function *in vitro*

**Figure 5:**
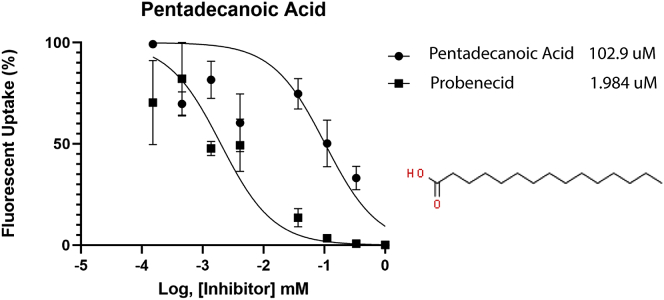
**Pentadecanoic acid inhibits OAT1 transport.** Pentadecanoic acid (chemical structure shown) competitively inhibited the OAT1-mediated uptake of 6-carboxyfluorescein in hOAT1 overexpressing HEK293 cells. In a separate experiment, probenecid inhibited OAT1-mediated uptake, as well.

Previous results from our group and others have shown that lipids of several classes, including prostaglandins, dicarboxylic acids, and short-chain fatty acids, directly interact with OAT1 *in vitro* (Table 1). To further explore this possibility, we performed a competitive inhibition assay between pentadecanoic acid, which was among the elevated lipids, and 6-carboxyfluorescein using hOAT1-overexpressing cells. Pentadecanoic acid had an IC_50_ value of 102.9 μM (Fig. 5), demonstrating that odd-length long-chain fatty acids have an impact on OAT1 transport. In addition, although not listed as lipids by the metabolomics software, several short-chain fatty acid derivatives, such as beta-hydroxyisovalerate and 2-hydroxybutyrate, were also elevated in the serum of the knockout mice, which is consistent with past characterizations of the knockout mice (10). Taken together, the previous and present *in vitro* transport data indicate that a wide variety of lipids in the classes elevated in the *Oat1* KO can interact directly with the OAT1 transporter.

**Table 1:** *In vitro* support for lipid interactions with OAT1

Metabolite	Ki (μM)	IC_50_ (μM)	Inhibition	Reference
Bile Acids				
Taurocholate	2800			(10)
Short-Chain Fatty Acids				
3-Hydroxybutyrate	3220			(54)
β-Hydroxybutyrate	1023			(25)
Butyrate	3500			(55)
Propionate	8083			(54)
Prostaglandins				
Prostaglandin E1	12			(24)
Prostaglandin E2	3.4			(55)
Prostaglandin F2α	0.57			(56)
Long-Chain Fatty Acids				
16-Hydroxy-hexadecanoic acid	13			(10)
Hexadecanedioate			30–70% Inhibition	(57)
Palmitoleic acid	200			(24)
Pentadecanoic acid		102.9		This study
Tetradecanedioate			30–70% Inhibition	(57)
Medium-Chain Fatty Acids				
Adipic acid	0.41			(55)
Heptanoate	16.7			(55)
Hexanoate	38			(55)
Octanoate	5.41			(58)
Suberate	34.19			(55)
Dicarboxylic Acids				
Fumarate	610			(55)
Glutarate	6.7			(55)

### *In vivo* metabolomics overlap with Recon3D predictions

Finally, we reconsidered all the metabolomics data together (*Oat1* knockout and probenecid treated) in light of the Recon3D metabolic reconstruction presented at the beginning of the Results. As mentioned earlier, the reconstruction was based on previous transcriptomics and untargeted metabolomics data from a decade ago. Thus, the Recon3D metabolic reconstruction results can also be viewed as potential predictions of altered biochemical reactions and metabolites, particularly in the context of lipid metabolites, given that Recon3D accounts for 600 more lipids than earlier versions.

The metabolic reconstructions provided interpretations of the altered flux states resulting from the loss of function of OAT1. These alterations may result from metabolites that directly interact with OAT1 as well as indirectly (through secondary shifts in metabolite availability). Of the 312 metabolites predicted to be altered in the model, 39 were measured in at least one of the animal experiments. Among those, several were indeed altered in an experiment and represented the major classes of lipids, including PUFAs, eicosanoids, LCFAs, and eicosanoids (Table 2). Thus, even though the constraining of the Recon3D model was limited, the metabolic reconstruction predicted most of the major altered lipid classes.

**Table 2:** Fourteen metabolites were altered in the metabolic reconstruction using *Oat1* KO data and in at least one of the *in vivo* serum metabolomics experiments

Metabolite	HMDB ID	Experiment
Taurocholate	HMDB00036	Knockout
Palmitate (16:0)	HMDB00220	Drug treated
Hexadecadienoate (16:2n6)	HMDB00477	Knockout
Stearoylcarnitine (C18)	HMDB00848	Knockout
Tauroursodeoxycholate	HMDB00874	Knockout
Taurodeoxycholate	HMDB00896	Knockout
LTB4	HMDB01085	Drug treated
PGE2	HMDB01220	Drug treated
Erucate (22:1n9)	HMDB02068	Knockout
Linolenate [alpha or gamma; (18:3n3 or 6)]	HMDB03073	Both
15(S)-HETrE	HMDB05045	Drug treated
Dihomo-linoleate (20:2n6)	HMDB05060	Knockout
5-HEPE	HMDB05081	Drug treated
5S-HETE	HMDB11134	Drug treated

## Discussion

In this work, we applied several different systems biology, wet lab, and computational strategies to clarify metabolic pathways and metabolites that are regulated or modulated by OAT1. We used genome-scale metabolic reconstructions to leverage transcriptomic and metabolomics data from *Oat1* KO and wildtype animals to create a systems biology model of OAT1-dependent biochemical reactions and the metabolites involved. The model predicted that several lipid-related pathways and metabolites were altered. The model predictions were supported with global targeted metabolomics on the serum of *Oat1* KO mice that included 342 lipids spanning 37 unique subpathways. It is surprising that over 35% of these metabolites were altered by the absence of OAT1. To date, only a few lipids have been linked to OAT1 (23, 24). Here, we have added to the potential list of lipids transported by OAT1 in addition to those we have tested here and previously. Furthermore, the knockout mice metabolomics and systems biology predictions were supported by targeted metabolomics for eicosanoids and free fatty acids in the serum of rats treated with probenecid.

Taken together, the data emphasize the importance of OAT1 in regulating systemic lipid metabolism, potential lipid-mediated signaling, and even intracellular signaling within the proximal tubule. Many fatty acids, which are organic anions with hydrocarbon chains of various lengths, are now appreciated not only for their central role in energy metabolism but also for their role in signaling *via*, for example, binding of G-protein-coupled receptors and nuclear receptors (27, 28) (Table 3). It was long believed that fatty acids passively diffused across the membrane, but increasing evidence suggests this is unlikely (38, 39). The combined action of multi-specific transporters, as well as other mono- or oligo-specific transporters, may represent a route of entry and exit for fatty acids into the renal proximal tubule cell. The increased availability in the blood also makes it possible that these metabolites will impact other organs. PUFAs and free fatty acids are able to activate nuclear receptors and G-protein coupled receptor like NF-κB, PPAR, and FFAR (40, 41). PUFAs are also the precursors to eicosanoids, a family of locally acting bioactive lipids that have been linked to a number of diseases (42). Little is known about the elevated eicosanoids’ function, but PGB2 has been suggested to activate tyrosine kinases and even translocate NF-κB to the nucleus (43). DAGs, widely recognized as PKC activators, can also be degraded to release free fatty acids, which have independent signaling roles in different tissues (27).

**Table 3:** Signaling capacity of some elevated metabolites in the knockout or probenecid-treated animals

Lipid	G-protein coupled receptor or nuclear receptor ligand (59, 60, 61)
Myristic acid (14:0)	✔
Pentadecanoate (15:0)	✔
Palmitic acid (16:0)	✔
Palmitoleic acid (16:1)	✔
Linoleate (18:2n6)	✔
Linolenate [alpha or gamma; (18:3n3 or 6)]	✔
Gamma-linolenic acid (18:3n6)	✔
Stearidonate (18:4n3)	✔
Nonadecanoate (19:0)	✔
Arachidate (20:0)	✔
Dihomo-linoleate (20:2n6)	✔
Eicosapentaenoate (EPA; 20:5n3)	✔
Docosatrienoate (22:3n3)	✔

Although the elevated metabolites have a clear mechanism for their systemic increases—with the transporter they depend on for entry into the proximal tubule cell blocked by probenecid or absent due to gene disruption—there were also metabolites that were significantly decreased. These included bile acids, ceramides, and several eicosanoids, which also carry important signaling roles. For example, bile acids are potent natural ligands for FXR, a nuclear receptor that regulates several drug handling genes (44). Tauro-beta-muricholate, a significantly decreased metabolite, is a gut microbe-derived secondary bile acid that serves as an FXR antagonist (45). It is possible that lower levels of systemic tauro-beta-muricholate can impact signaling by nuclear receptors in the liver and intestine. For both elevated and decreased metabolites, it is important to note that even small changes in systemic levels may lead to significant physiological effects.

The well-established multispecific nature of OAT1 makes it a potential source of DMIs and drug–drug interactions (DDIs), a major concern for regulatory agencies (46). The International Transporter Consortium and the US Food and Drug Administration have recommended that pharmaceutical developers test their products for interactions with seven drug transporters, OAT1 among them, because of the potential for DDIs when patients are taking multiple drugs (47). Although many DDIs involving these drug transporters have been analyzed, it is worth noting that a similar phenomenon—namely, DMIs with endogenous compounds—is possible (14). In this case, drugs and endogenous compounds compete for transporters leading to increased half-life of drugs or increased concentrations of the endogenous metabolites (many of which are signaling molecules and/or toxic at higher concentrations) (14). Here, we have identified lipids that may be implicated in DMIs.

Partial inhibition or competition for OAT1 by drugs may have metabolic consequences due to the various pathways that can be altered. Chronic drug treatment increases the risk of metabolic syndrome in part through altering lipid metabolism (29). Diuretics and antivirals, several of which are OAT1 substrates, are among the medications that can cause drug-induced metabolic syndrome (29, 48). Our knockout animals showed major alterations in lipid metabolism and a suggestion of lipid deposition in very aged livers. However, this finding should be viewed with caution, since only a few mice of this age were available. We emphasize that a much larger study of aged *Oat1* knockout mice would be required to statistically demonstrate whether or not lipid deposition is affected in the liver or other organs. In addition, some of the elevated eicosanoids (11,12-diHETrE and 20-COOH AA) in the probenecid-treated animals have been identified as potential biomarkers for diagnosis of nonalcoholic liver disease (49).

As systems biology models continue to improve, so too do the predictions they generate. Apart from advances in methodology, the Recon3D metabolic reconstruction presented here considers over 600 additional lipid metabolites and attendant biochemical reactions. New metabolites, including eicosanoids and fatty acids, were predicted to be altered and confirmed by metabolomics. This methodology can be applied to several drug transporters in the kidney, liver, intestine, and other organs that have a limited set of known metabolites amidst a wide range of potential substrates. Here, *in vivo* support is provided to define how a multispecific transporter, like OAT1, functions in systemic physiology. Although we have focused on the renal role of OAT1, it should also be noted that the global knockout may also affect the metabolism of other OAT1-expressing tissues. The chemoinformatic analysis can also be used to predict potential DMIs, as not every metabolite can be measured *via* metabolomics. With an increase in metabolomics-transporter knockout data for multiple SLC and ABC drug transporters, DMIs can be better modeled and prevented clinically, and drug-induced metabolic derangements can be better understood.

## Experimental procedures

### Animals

The UCSD Institutional Animal Care and Use Committee approved the experimental protocols. The animals were handled in accordance with the Institutional Guidelines on the Use of Live Animals for Research; all experiments involving the use of animals were also conducted in accordance with the Institutional Guidelines on the Use of Live Animals for Research.

The *Oat1* knockout mice in this study were described in a previous work (RRID:MGI:3798165) (34). To briefly recapitulate, mice were housed in a 12-h dark/light cycle with *ad libitum* access to standard mouse chow and water. Whole blood was collected from *Oat1* KO male mice (n = 5) and their wildtype controls (n = 5). Serum was extracted from the whole blood.

Adult male Sprague Dawley rats (n = 3) were acquired from Envigo-Harlan. The treated group was administered water-soluble probenecid (Invitrogen) in PBS at 200 mg/kg (in line with standard dosage) *via* intraperitoneal (i.p.) injection. The control group was treated with PBS (sham) *via* i.p. injection. Rats were sacrificed after 2 h (below the reported half-life of probenecid), and whole blood was collected. Serum was isolated and stored as unpooled samples at −80 °C until metabolomic analysis.

### Metabolomics analysis

Serum samples from mice were sent to Metabolon Inc for global targeted metabolomics as described (34). Missing data were filled with the lowest value from a mouse within the same group. Statistical comparisons were performed using Welch’s two-tailed *t* test, with a *p*-value ≤0.05 indicating statistical significance and a *p*-value of 0.05 < *p* ≤ 0.10 indicating a trend toward statistical significance. FDR-adjusted *p*-values and Cohen’s d were also calculated and are reported in Table S4.

Per Metabolon, enrichment was calculated for subpathways with over five metabolites using $\frac{\frac{k}{m}}{\frac{n-k}{N-m}},$ where *k* is the number of significantly altered metabolites in a subpathway, *m* is the number of metabolites in a subpathway, *n* is the number of significantly altered metabolites in the total dataset, and *N* is the number of measured metabolites in the total dataset.

Lipid analysis was performed at the UCSD Lipidomics Core (50). Targeted metabolomics analyses were performed separately for free fatty acids and eicosanoids. A total of 32 free fatty acids were identified, and 77 eicosanoids were identified. Statistical comparisons were performed using a Student’s *t* test.

### *In vitro* transport assays

Transport assays were carried out as described (24). OAT1-overexpressing HEK cells were maintained in fetal bovine serum supplemented with Dulbecco's modified Eagle's medium and 1% antibiotic (penicillin/streptomycin) in 5% CO_2_ at 37 °C. Cell media also contained blasticidin, a selective marker for OAT1-overexpressing cells. For transport assays, cells were plated and incubated for 24 h until grown to confluence. Competitive inhibition experiments were performed with a fixed concentration of 10 μM 6-carboxyfluorescein and a serial dilution of the probe substrate starting at 2 mM. Controls were carried out using 6-carboxyfluorescein and probenecid. Pentadecanoic acid was dissolved in ethanol at low concentration to prevent cell death. Pentadecanoic acid was added to the first column of a 96-well plate and a 3-fold dilution was carried out for the other columns. Cells were rinsed with ice-cold PBS three times, and fluorescence was measured with a fluorescent plate reader. Fluorescent values were normalized for each column, and the IC_50_ values were calculated using GraphPad Prism 9.

### Metabolic reconstructions

As detailed in Results, transcriptomic data from *Oat1* KO and wildtype kidneys (11) were used to generate condition-specific metabolic reconstruction models with Recon 3D (31). The models were constrained with previously acquired metabolomics data from the urine and plasma of *Oat1* KO mice (13). Differential mean flux states between wildtype and knockout conditions were then computed from the normalized sampled feasible flux states for F-test calculations at significance *p* < 0.001, with at least >2-fold change (or <0.5-fold change).

### Chemoinformatics, data visualizations, and machine learning analyses

The analysis pipeline is largely described in a previous publication (34). In brief, over 30 molecular and physiochemical properties were calculated for the lipid molecules with Pubchem IDs altered in the *Oat1* KO. Using Orange, a machine learning software based in the Python library Scikit-learn, a variety of methods, metrics, and visualizations were employed to further select features (51). These included information gain, Freeviz (52), and principal components analysis. SciPy python packages were also used in the analysis (53). The features were ultimately narrowed down to six by removing highly correlated features with similar information (molecular area, molecular volume) and traditional visualizations. Since there were many more elevated than decreased lipids, for machine learning analyses, the groups were balanced. A combination of downsampling and upsampling methods were employed, and the “leave one out” method was used for validation. Some of the analysis was also separately confirmed with hand-coded Python using the Pandas, Seaborn, and Scikit-learn libraries, but only the Orange results are reported here.

### Oil red O staining and image analysis

Mouse livers from male *Oat1* KO or wildtype mice aged 24 months were harvested, placed in 10% neutral buffer formalin for 24 h, moved to 30% sucrose for 24 h, and frozen in Optimal Cutting Temperature with isopentane in dry ice. Oil Red O and H&E staining were performed by the Mouse Histology Core at UC San Diego. Images were analyzed with FIJI using the Color Transformer plugin. Each image had its brightness and contrast adjusted to enable comparisons between images. Images were converted to the YIQ color space, and analyses were carried out on the I channel images. Images were converted to 8 bit, and a threshold for each image was set to distinguish the lipid droplets from the rest of the slide. The Analyze Particles feature in FIJI was then used to measure the total area covered by the lipid droplets, the number of droplets, and the average size of the droplets. Statistical comparisons were made using a one-tailed Student’s *t* test assuming equal variance.

## Data availability

All data are contained within the article. Transcriptomic data are available upon request from snigam@health.ucsd.edu.

## Supporting information

This article contains supporting information.

## Conflict of interest

The authors declare that they have no conflicts of interest with the contents of this article.
