# Supplementary material for: A key role for the transporter OAT1 in systemic lipid metabolism
Source: J Biol Chem. 2021 Mar 27;296:100603. doi: 10.1016/j.jbc.2021.100603 (PMC8102404; doi:10.1016/j.jbc.2021.100603)
Supplement: Supplemental Figures S1 and S2 [file mmc1.pdf]

Supplementary Information

A key role for the transporter OAT1 in systemic lipid metabolism

Jeffry C. Granados, Anisha K. Nigam, Kevin T. Bush, Neema Jamshidi, Sanjay K. Nigam

		Predicted		
		**DOWN**	**UP**	**∑**
Actual	**DOWN**	42	4	**46**
	**UP**	7	39	**46**
	**∑**	**49**	**43**	**92**

Supplementary Figure S1. The confusion matrix from the random forest model. The confusion matrix shows the number of incorrectly classified lipids.

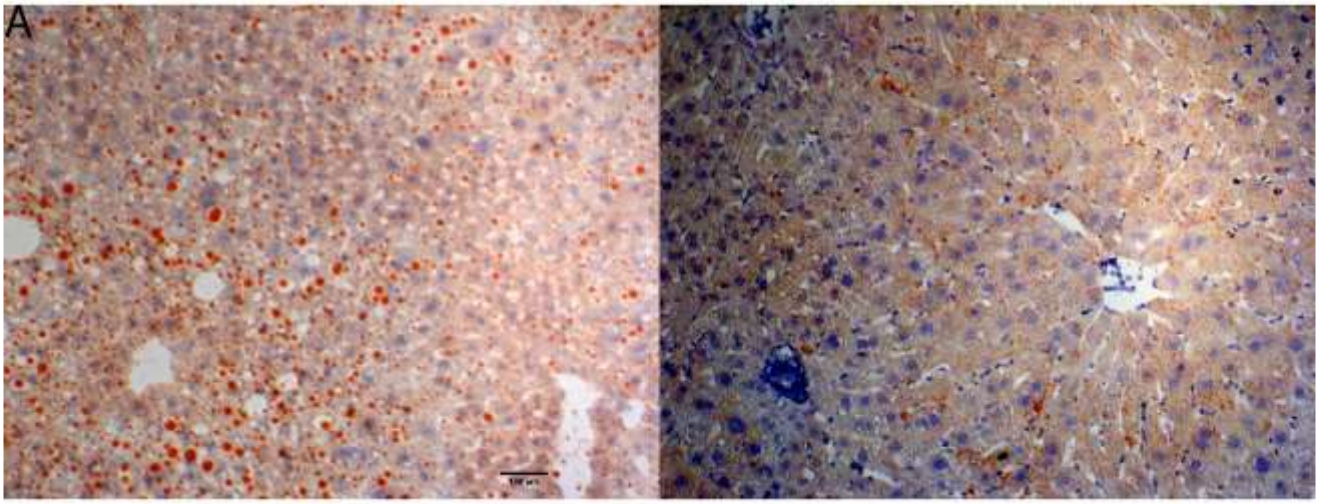

B

	Total area covered by lipid droplets (pixels)	% Area covered by lipid droplets
WT Average	16505.4	1.185
WT Standard Error	3082.563375	0.221362147
Oat1 KO Average	29166.6	2.0944
Oat1 KO Standard Error	15498.56731	1.112918577
P Value	0.223069608	0.223015418

Supplementary Figure S2. Some *Oat1* knockout mice show increased lipid droplets when compared to age-matched wild types. A) Representative image of 24-month-old *Oat1* knockout and wild type mice Oil Red O-stained livers. We observed a trend indicating that *Oat1* knockout mice show more lipid droplets. Scale bar = 100 um. **B)** The area covered by the lipid droplets was measured for the *Oat1* knockouts versus the wild types (n= 5 for each group).
